# A Visual Approach for the SARS (Severe Acute Respiratory Syndrome) Outbreak Data Analysis

**DOI:** 10.3390/ijerph17113973

**Published:** 2020-06-03

**Authors:** Jie Hua, Guohua Wang, Maolin Huang, Shuyang Hua, Shuanghe Yang

**Affiliations:** 1 Faculty of Information Engineering, Shaoyang University, Shaoyang 422000, China; ysh_ysh2020@outlook.com; 2 School of Software Engineering, South China University of Technology, Guangzhou 510006, China; 3 Faculty of Engineering and IT, University of Technology Sydney, Sydney 2007, Australia; mao.huang@uts.edu.au; 4 Faculty of Engineering, University of Sydney, Sydney 2007, Australia; shua6688@uni.sydney.edu.au

**Keywords:** visual analysis, graph visualisation, graph drawing, SARS, coronavirus, COVID-19

## Abstract

Virus outbreaks are threats to humanity, and coronaviruses are the latest of many epidemics in the last few decades in the world. SARS-CoV (Severe Acute Respiratory Syndrome Associated Coronavirus) is a member of the coronavirus family, so its study is useful for relevant virus data research. In this work, we conduct a proposed approach that is non-medical/clinical, generate graphs from five features of the SARS outbreak data in five countries and regions, and offer insights from a visual analysis perspective. The results show that prevention measures such as quarantine are the most common control policies used, and areas with strict measures did have fewer peak period days; for instance, Hong Kong handled the outbreak better than other areas. Data conflict issues found with this approach are discussed as well. Visual analysis is also proved to be a useful technique to present the SARS outbreak data at this stage; furthermore, we are proceeding to apply a similar methodology with more features to future COVID-19 research from a visual analysis perfective.

## 1. Introduction

The recent COVID-19 outbreak has infected 216 countries, areas or territories in the world as of 29 May/2020 [1]; this has brought closely into our sight the SARS outbreak of 2003, when there were a total of 8096 cases reported, including 774 deaths in 29 countries between 01 November/2002 to 31 July/2003 [2]. SARS-CoV and SARS-CoV-2 (Severe Acute Respiratory Syndrome Coronavirus 2) are 82% similar in their genome sequences; SARS-CoV-2 is also 96% identical at the whole-genome level to a bat coronavirus [3]. Since they all belong to the coronavirus family [4,5], similar prevention measures [6] have been applied to both as well. To tackle this worldwide health crisis, medical/clinical research is surely essential, along with studies from other various perspectives, such as virus data analysis, etc., which may also assist in offering deeper insights.

In 2003, WHO (World Health Organization) finalised a consensus document, which contained details of all infected areas, where the evidence has confirmed the efficacy of traditional public health measures, which include early case identification and isolation, vigorous contact tracing, voluntary home quarantine of close contacts for the duration of the incubation period, and public information and education to encourage prompt reporting of symptoms [7]. In relation to prevention measures, essential infection controls include isolation, contact tracing, school closure, less travel, avoiding crowded places, sanitising and wearing a mask, etc. [6,7,8,9,10,11,12,13,14]; detailed analysis of factors including age, gender, mortality rate, HCW (Health Care Worker) rate and more susceptible places has been conducted in some countries and regions as well [7,11,13,15,16,17]. Related works on effective data collection for non-medical research have been published [18,19]; besides, Xu et al. (2020) have explained the coronavirus family, which includes SARS, MERS (Middle East respiratory syndrome) and SARS-CoV-2, etc. They also carried out a systematic comparison between SARS and SARS-CoV-2, determined that treatments such as isolation, antiviral and symptomatic treatments are effective methods for both viruses [6]. Most of the studies above utilise visualisation tools to finalise outcomes.

In this study, there are multiple data types ranging from date type and numeric type to timeline event type; therefore, being able to process and grasp insights from these complex data has become a key challenge. Additionally, when this work is for non- medical and/or clinical research purposes, we need to keep it comprehensible and easy-to-understand for readers, especially those who do not have relevant expertise. On the other hand, data visualisations are common techniques that use graphs to offer rich representation structures for bringing insights into complex data; besides, they come with easy-to-understand forms, and finalise outcomes with evidence for decision-making purposes [20]. They have been exploited in fields such as the financial sector [20,21,22,23,24], social network analysis [25,26,27,28,29], virology research [6,7,8,9,10,11,12,15,16,27], etc., to effectively discover large and complex datasets. Some techniques involved in exiting related works include line charts [8,9,15,16,30], bar charts [6,7,8,9,11,12,15], geographic visualisations [6,15] and parallel coordinate plots [8]. Most of those related existing works provide clear and effective visual outcomes. Applying visualisation methods enables visual summary statistics, which can be used to tackle challenges such as displaying increasing amounts of dense information with multiple data attributes in a human-readable manner, hence, to better inform public health and treatment decisions [31]. 

In this visual approach, we apply a line chart, a bar chart, a geographic visualisation and a timeline. The line/bar chart component is capable of displaying multiple series of data on a chart. Geographic visualisation provides related information in a captivating and intuitive way, to provide more insight into the overall structure of a dataset and to visually inspect what geographic patterns arise in maps [32,33]. Timeline visualisation is an approach to visualise temporal data; it provides insights into the joint work by presenting all features and relatively temporal information, it reduces crossings and overlaps of saccade lines [34,35,36].

To the best of our knowledge, most existing studies [8,9,10,11,12,13,14,15,16,17,18,31] of the SARS outbreak are processed in their medical/clinical aspects, along with some visualisation tools to offer views on particular features; there, a few studies have been done from a total data analytics aspect, to provide a ‘big picture’ and a potential pattern to discover for related virus data analysis. In this work, we address the SARS raw data visual analysis, and try to extract deeper insights from the SARS data on the five most affected countries and regions. This study is not related to medical/clinical research; it is purely based on data analysis methods. Here, during the SARS outbreak in 2003, our hypotheses are finalised based on infection case features and outbreak facts from exiting works [6,7,8,9,10,11,12,13,14,15,16,17,18,19], since other features such as human behaviour, area features, patient details, etc., are hard to fetch for all areas in 2003.

- H1: there are case features (such as similar peak period, prevention measures) in common in five areas.
- H2: detailed outbreak facts (such as mortality rate, outbreak duration) differ in five areas.
- H3: a visual approach can assist readers (not experts in relevant fields) in getting a big picture effectively.
- H4: it is possible to work out a good reference sample for SARS lifecycle analysis, as well as effective prevention measures.

A potential hypothesis, which is that there will be similar patterns in the SARS-CoV-2 data analysis, is not included in this work; it will be studied in our future work.

Our work aims to provide non- medical and/or clinical techniques capable of analysing the SARS outbreak and to extend these for similar virus data analytics, such as the COVID-19 in the future—hence, to offer patterns for references in decision-making and/or trend prediction in related fields. In this article, date descriptions without the year all indicate 2003; for the description of countries and regions, China means Mainland China; Hong Kong represents Hong Kong, China; Taiwan indicates Taiwan, China; Canada here only means Toronto, Canada.

The rest of this article is organised into several parts. In Section 2, relevant data and its processing step details are given; we also introduce related methods such as graph drawing tools, methods and features involved in experiments. In Section 3, we offer visual results from five aspects, as well as an overview dashboard. We summarise statistical data and discuss issues found in Section 4. Eventually, we conclude our work and discuss future research in Section 5.

## 2. Materials and Methods 

In this section, we introduce the workflow of our research, as well as raw data, data processing, and relevant graph visualisation tools in the SARS data analysis. 

### 2.1. Materials

#### 2.1.1. Data Collection

We downloaded and collected two different types of raw data for experiments:

1. Case Data

Data including every day’s case details, such as infected case number, cured case number, death number, etc., were downloaded from the WHO [37].

2. Events Data

Data including major events such as revision of the WHO’s list of areas with local transmission, different areas’ lockdown measures, etc., were collected from the WHO at [38], along with the Singapore government website at [39].

Details of raw data downloaded/collected are shown in Table 1, in which case data range between 17 March and 11 July, events data range between 16 November/2002 and 15 July/2003.

#### 2.1.2. Data Processing

All raw data collected have been cleansed and formatted. One issue was met during the data processing stage: many of the case data were not reported to WHO. We figured out a simple way to fulfil the data on the non-reported days, in detail, to make the values continuous and distributed evenly during the non-reported days. Suppose *d_s_* is the case number on the day before reports to WHO stops, *d_r_* is the case number on the day reports to WHO restarts, *D_e_* are the days {*d_1_*,…,*d_e_*} which have no reports to WHO, and *e* indicates the number of days, then

(1) $$d_{n(n\in e)}=d_{s}+n\times \frac{dr-ds}{e+1}$$

represents the case number on day *n*. The same method was also applied to mortality number, cured number features, etc.

Eventually, for the case data, 117 rows of records with 26 columns for each row have been kept for further experiments; here, columns present data attributes such as date, infected number, death number and cured number, etc. For the events data, 29 major records were saved as well (events on the same days were merged into one record). 

#### 2.1.3. Graph Generation Tool

Tableau Public [40] is a common platform for visualisation research and development purposes; it comes with rich features to create interactive data visualisation outcomes, and it is the free version of the paid Tableau software [41,42]. In the experiments, we use finalised data files as inputs and Tableau Public as a tool to generate graphs and dashboards, in detail, including line charts, stacked bars, maps and timelines to provide visual results. These come with tables to present complex data, since they are easy to implement and capable of displaying increasing amounts of dense information in a human-readable manner [19].

### 2.2. Methods

#### 2.2.1. Feature Selection

Some common features are taken into account in relevant studies, such as infected case number/rate [6,7,8,9,10,11,12,13,14,15,16,17,30], mortality number/rate [7,8,9,10,12,13,15,16,17,30], cured number/rate [6,7,8,9,10,13,15,30], HCW infected number/rate [7,8,13], patient gender [6,7,8,9,10,11,13,15,16,17], patient age [6,7,8,9,10,11,13,14,15,16,17,30], prevention measures [6,7,10,11,12,13,14,15] and event timeline [6], etc. In this work, we selected five major features to generate graphs for assisting in analysing the SARS outbreak data: daily existing infected case number, to-date mortality rate, to-date cured rate, daily changing rate of infected case number, and events timeline.

Facts from this work that differ from previous studies are as follows:

1. We apply the daily changing rate of infected case number to offer another angle of view on the virus spreading trends, such as how fast the outbreak is between every two continuous days.

Suppose the infected case numbers on two continuous days are n_i_ and n_i+1_, and raw data are collected from day *1* to day *k*; then, the changing rate between those two days is r_ni_ = (n_i+1_ – n_i_) / n_i_. Therefore, the changing rates array is R= {r_n_*_1_*,r_n_*_2_*,…,r_nk-1_}; all input data in this feature’s experiments have been processed in the data processing step mentioned in Section 2.1.2.

2. We utilise the events timeline feature to bridge the virus outbreak and major events (events such as revision of the WHO’s list of areas with local transmission, quarantine measures applied, etc.); hence, we try to detect the impacts of applying prevention measures.

This work’s experiments are also not age- or gender-standardised, neither are HCW infection details; we only mention gender and HCW differences in patients in Section 3.3. In this article, prevention measures mainly indicate school closure, since there were no strict lockdown rules in the SARS outbreak in 2003 [6,7,10,11,12,13,14,15].

#### 2.2.2. Procedure

Based on the raw data finalised from the data processing steps, the proposed approach uses Tableau tools to generate graphs from five features, combined with a dashboard; then, it compares the results of five countries and regions to bring out insights into all the data involved. The steps included in the workflow of this study are shown below.

1. Collecting raw data from multiple sources.
2. Data filtering and formatting, such as removing duplicated data, adding data entries on unreported days, then formatting and importing into data files.
3. Comparing visual results via data values and observation.
4. Concluding data for key nodes (values on particular days) and issues.
5. Discussion.

## 3. Results

The following results are presented with five features: daily existing infected case number, to-date mortality rate, to-date cured rate, daily changing rate of infected case number, and events timeline. An overview dashboard is given as well. 

We also applied a t-test to determine if there was a significant difference between the means of two datasets: Excel’s t-test. A two-samples t-test assuming unequal variances was used on the daily changing rate of infected case number, to-date cured rate and to-date mortality rate, since unequal variances are less problematic if data sample sizes are similar [43]. The *p*-value is the probability of obtaining test results at least as extreme as the results observed during the test. Alpha is a chosen significance level in the experiments (alpha = 0.05 in this study); a null hypothesis is that there is no significant difference between two data samples [44]. In experiments, the p-value is compared to alpha to determine if the null hypothesis can be rejected [45].

- If *p* > alpha: Accept the null hypothesis that the means are equal.
- If *p* ≤ alpha: Reject the null hypothesis that the means are equal.

In experiments, these null hypotheses related to the t-test in Section 3.2,Section 3.3 and Section 3.4 are that relevant rates in different areas are similar.

### 3.1. Daily Existing Infected Case Number

**Figure 1:**
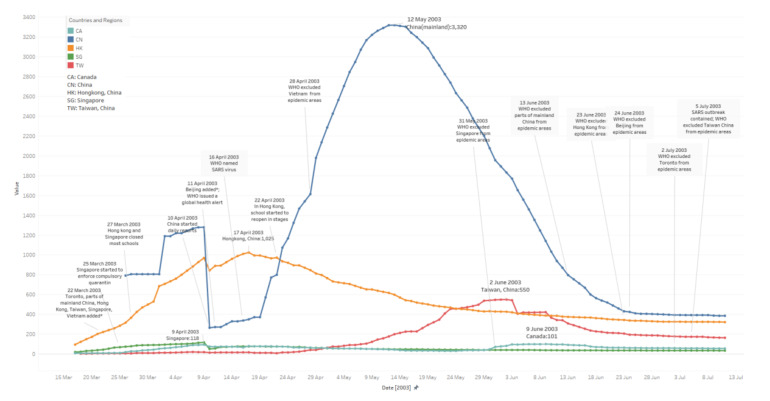
Daily existing infected case number trends of five countries and regions. (17 March–11 July).

In Figure 1, lines indicate existing SARS infected case numbers trends in the specific period; all trends in the line chart are similar except China’s. During the outbreak, the virus begins to infect more people. Normally, when it reaches the peak, the existing case number starts to decrease until it stabilises. Besides, in this figure, major events are added to help clarify the timeline of the entire SARS outbreak; a detailed events timeline is offered in Section 3.5.

From Figure 1, the peak period is calculated for days with new daily case numbers greater than or equal to the relevant median values. Some facts are as follows:

- China: The median value here is 1155. The peak period lasts 68 days, from 2 April to 08 June. It reaches a peak with 3320 cases on 12 May. From the very beginning, 26 March to 09 April, the trends in the figure are messy. Raw data is not accurate, which might be because potential patient details were not fully tested or reported, etc., until 10 April.
- Hong Kong: The median value here is 450. The peak period lasts 59 days, from 29 March to 26 May. It reaches a peak with 1025 cases on 17 April. Its symmetry before and after the peak appears better than for other areas via observation (before-peak period: 30 May–17 April; after-peak period: 17 April–04 May; Singapore and Canada are not included due to fewer cases).
- Taiwan: The median value here is 168. The peak period lasts 68 days from 12 May to 08 July. It reaches a peak with 550 cases on 02 June; the trend in the figure jumps several times. From digging into the raw data we collected, potential reasons may include misdiagnosis, etc.
- Singapore: The median value here is 45. The peak period lasts 60 days from 23 March to 21 May.
- Canada: The median value here is 60. The peak period lasts 91 days form 03 April to 02 July.

Since the median value is a statistical measure inherently robust to the presence of outliers [46], we apply median values to estimate the peak periods of each area in the dataset, which are measured between days when infected cases increase to reach the median number, and when all daily infected case number stabilises to below the median number, accordingly.

### 3.2. To-Date Mortality Rate

**Figure 2:**
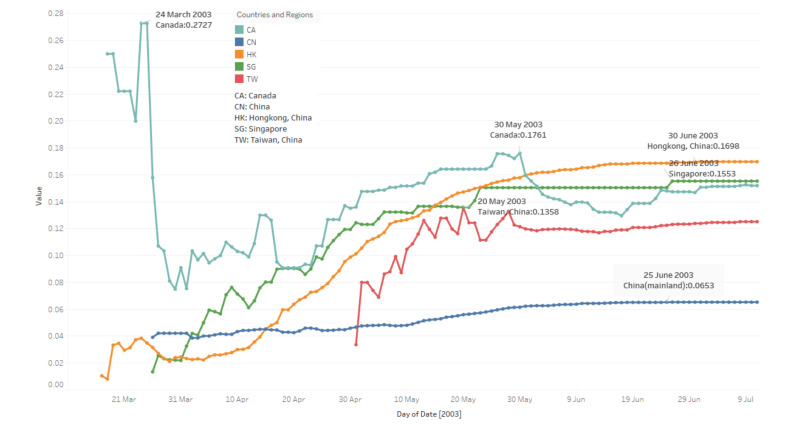
To-date mortality rate trends of five countries and regions. (17 March–11 July).

In Figure 2, lines indicate present to-date mortality rate trends in the specific period. Most likely, at the beginning of the outbreak, rates jump up and down until they reach the peak, then stabilise. Rates are calculated from the day when the first death cases are reported, which does not mean there is no virus outbreak before that day. This feature’s results are supposed to be a subset of the daily existing infected case number results, so, from this point of view, the stabilizing date presents the day when the mortality rates become steady.

From Figure 2, based on median values which indicate that mortality rates stabilise in each area, some facts are as follows:

- China: The median value is 0.0549; mortality rates tend to be steady from 19 May.
- Hong Kong: The median value is 0.1337; mortality rates tend to be stabilizing from 14 May.
- Taiwan: The median value is 0.1198; mortality rates tend to be stable from 13 May.
- Singapore: The median value is 0.1366; mortality rates stabilise from 12 May.
- Canada: The median value is 0.1471; mortality rates remain steady from 02 May.

In Figure 2, Hong Kong and China’s trends change smoothly; China has the smallest median mortality rate, followed by Taiwan, Singapore, Hong Kong and Canada. Mortality rates all reach their peaks in May in five areas. Besides, we processed a t-test between every two areas’ mortality rates; the results in Table 2 show that Hong Kong, Singapore and Taiwan have similar rates. The bold font in Table 2, 3 and 4 present the *p*-Value larger than the Alpha value.

### 3.3. To-Date Cured Rate

**Figure 3:**
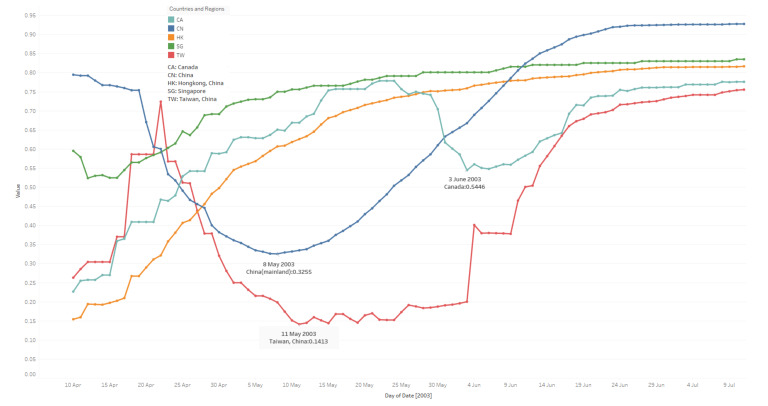
To-date cured rate trends of five countries and regions. (10 April–11 July).

In Figure 3, lines indicate present to-date cured rate trends for a specific period. At the beginning of the outbreak, rates are not stable, especially in China and Taiwan. Rates are calculated from the day when there are cured cases reported, which does not necessarily mean things are worse before the days, since recovery needs time. This feature’s results are supposed to be a subset of the daily existing infected case number results, so, from this point of view, the stabilizing date presents the day when the cured rates have remained steady since then.

From Figure 3, based on median values which indicate that cured rates stabilise of each area, some facts are as follows:

- China: The median value is 0. 7074; cured rates tend to be steady from 05 June.
- Hong Kong: The median value is 0. 7393; cured rates tend to be stabilizing from 26 May.
- Taiwan: The median value is 0.3791; cured rates tend to be stable from 08 June.
- Singapore: The median value is 0.7913; cured rates stabilise from 23 May.
- Canada: The median value is 0.6507; cured rates remain steady from 17 June. (The cured rates drop a lot between 31 May to 16 June.)

In Figure 3, Hong Kong and Singapore’s trends change smoothly and keep rising, potentially indicating that case data are reported punctually and integrally from those two areas, and the local governments handle the virus outbreaks well. On the contrary, in China and Taiwan, the cured rates seem to be good at the beginning and keep decreasing to reach 0.33 and 0.14 on 08 May and 11 May, then start rising, and take around five weeks to finally stabilise, potentially caused by cases unreported, misdiagnosis, etc. (Since China started daily reports from 10 April, previous potential cases might not be reported to WHO; based on data collected related to Taiwan, the infected case number and cured number change frequently; e.g. on 07 April, the total infected case number is 21, yet, on 8 April, it is 19; see details at [47] and [48]. Another interesting thing is that trends in Canada tend to jump a lot, with the cured rates getting worse when the other four areas get better; Toronto was put on the WHO’s list of areas with local transmission twice. Reasons remain unclear; this could be caused by unstrict prevention measures, but there is no clear data to support it at this stage. We also processed a t-test between every two areas’ cured rates; results in Table 3 show that China, Canada and Hong Kong have similar rates.

### 3.4. Daily Changing Rate of Infected Case Number 

**Figure 4:**
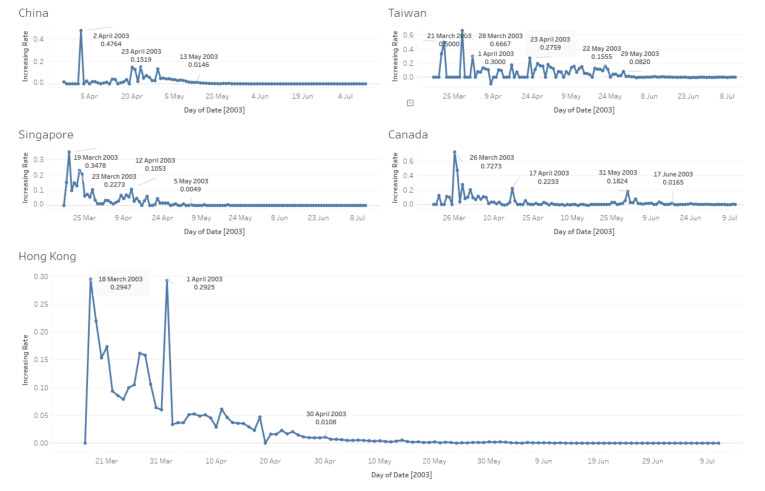
Daily changing rate of infected case numbers of five countries and regions. (18 March–11 July).

In Figure 4, lines indicate trends in changing rate between every two continuous days in a specific period. In the early stages of the SARS outbreak, changing rates vary a lot, especially in Taiwan and Canada. Hong Kong and Singapore tend to stabilise on 30 April and 5 May, before the other three areas.

From Figure 4, based on median values which indicate that daily changing rates of infected case number stabilise in each area, some facts are as follows:

- China: The median value is 0.00019; rates tend to be steady from 30 May.
- Hong Kong: The median value is 0.00236; rates tend to be stabilizing from 16 May.
- Taiwan: The median value is 0.00144; rates tend to be stable from 03 June.
- Singapore: The median value is 0; rates stabilise from 20 May.
- Canada: The median value is 0; rates remain steady from 11 July. (The rates go back and forth in June and July.)

Besides, we processed t-tests between every two areas’ daily changing rates; results in Table 4 show that China, Canada, Hong Kong and Singapore have similar rates.

### 3.5. Events Timeline

**Figure 5:**
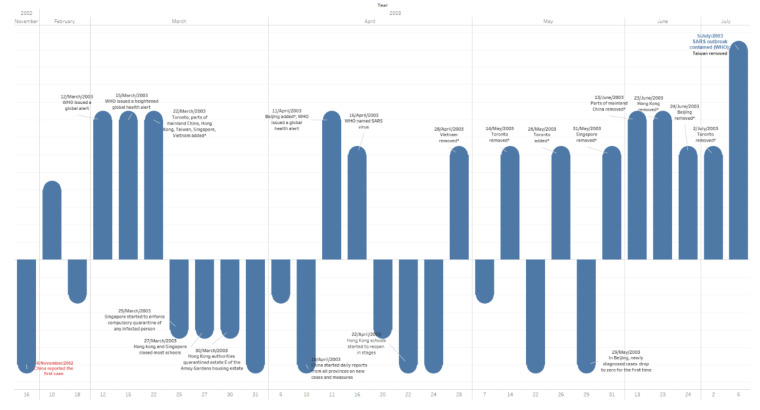
Major events timeline during the SARS outbreak. (16 November 2002–5 July2003).

Figure 5 presents the timeline of the major events during the SARS outbreak. Table 5 shows all events we collect and consider in experiments. “Weight” in Table 2 indicates the importance of the related event; basically, WHO’s announcements are normally more important, weighted at 4, such as issuing a global alert, revising the list of epidemic areas, etc. Local areas events’ weights range from 1 to 3, depending on their details; the most remarkable event here is that the WHO announced that the SARS outbreak was contained, which is weighted at 6. The height of bars in Figure 5 show the weight of each event. Some facts are below; the list here is the WHO’s list of epidemic areas.

- China was put on the list on 22 March; reached its peak on 12 May; and was removed from the list between 13 June and 24 June. (Schools in Beijing were closed on 24 April and reopened in stages on 22 May, but some were closed for another month [1]. Beijing was on the list between 11 April to 24 June; hence, there were 28 days school closure in Beijing. It was on the list for 74 days, but most areas in China were put on the list between 22 March and 13 June, for 83 days in total.)
- Hong Kong was put on the list on 22 March; schools were closed on 27 March; it reached the peak on 17 April; things were getting better from 22 April, when schools started to reopen in stages; it was removed from the list on 23 June; and there were 26 days school closure. It was on the list for 93 days.
- Taiwan was put on the list on 22 March; reached its peak on 02 June; and was removed from the list on 05 July. There was no school closure. It was on the list for 105 days.
- Singapore was put on the list on 22 March; relevant quarantine started on 25 March, schools were closed on 27 March; things were getting better from 09 April, when schools started to reopen in stages; it was removed from the list on 31 May; there was 13 days of school closure; and it was on the list for 70 days.
- Canada was put on the list on 22 March; it reached its peak on 09 June; it was removed from the list on 02 July; there was no school closure (several schools did close, yet no strict closure measures); and it was on the list for 102 days.

School closure periods in the facts above are all calculated from the first day of closure to the first day of any school reopened.

### 3.6. Overview Dashboard

**Figure 6:**
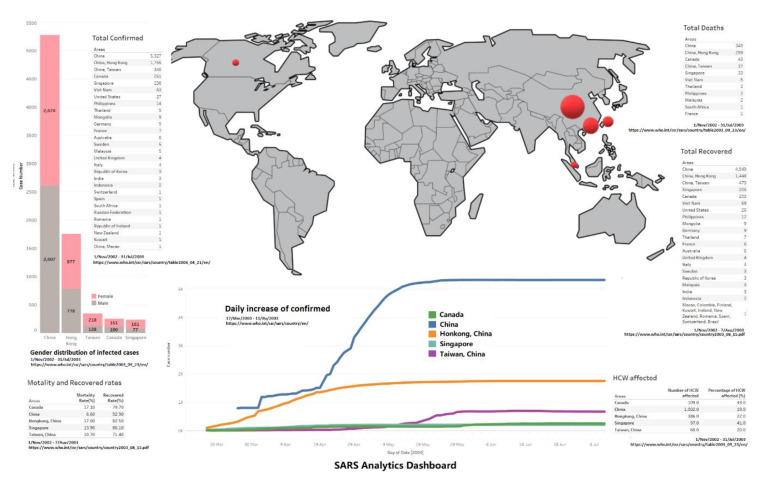
The dashboard of the SARS outbreak analytics. (This involves different periods, see details in the figure.)

In Figure 6, we finalise a dashboard to present the status of the SAR outbreak in 2003, using a map, line chart, stacked bar chart and table to present an overview, which includes total infected case number and its gender distribution, cured number/rate, death number/rate and HCW infected rate etc. From this figure, some facts are:

- Females seem more likely to get infected compared to male patients in all five areas, the female/male ratios of case numbers are 1.0257 (China), 1.269 (Hong Kong), 1.690 (Taiwan), 1.610 (Canada) and 2.090 (Singapore) (data involved till 31 July); this has been discovered in existing work [6]. However, another interesting thing which needs to be addressed is that male patients have a worse outcome than females in all age groups in Hong Kong [7]; there is no further data on gender infection results from other areas at the WHO, so we cannot conclude if the Hong Kong case is in particular or not.
- China has the highest cured rate and lowest mortality rate, but the trends of daily existing infected case number, to-date cured rate and daily changing rate of infected case number jump up and down a lot, and take a longer time to stabilise compared to Hong Kong and Singapore; those facts conflict, and might be because data was not fully reported until 10 April. Other than China, Hong Kong and Singapore show better outcomes on cured rate and mortality rate.
- Regarding the HCW infected rate, Canada and Singapore both report more than 40%; hospitals were struggling during the SARS outbreak.

## 4. Discussion

From all the results in Section 3, we estimate peak periods and summarise other details in Table 4. In the 2003 SARS outbreak, peak periods lasted for around 60 days in Hong Kong, Singapore and Taiwan; they were longer in China and Canada. In most areas, local governments applied relevant lockdown measures such as school closures, especially in China, Hong Kong and Singapore, although only several schools were closed in Canada and there were no school closures in Taiwan at all. Mortality rates tend to be between 10% to 17% till 11 July; the worst is around 17% in Hong Kong and Canada, yet China’s is only 6.6%. Hong Kong and Singapore present good cured rates which are more than 82%, with China’s at 92.9%. Singapore and Canada have the highest HCW infected rates, which are more than 41%. (All data in experiments are before 05 July.) There are also some issues found:

- China has the most infected cases and deaths, yet the lowest mortality rate and HCW infected rate, since the first case was reported on 16 November/2002 in Guangdong, China, and continuous daily reporting started from 10 April/2003. Instead, there is a 145-day gap, leading to data integrity issues. Hence, the discussions related to China are estimated and not accurate. However, data integrity is a common issue for all data collected from all areas by the WHO, especially in the early stages of the SARS outbreak.
- There were no strict lockdown measures in 2003 in those five countries and regions. Major prevention measures include quarantine of infected patients and school closures, etc., yet school closures made very little difference to the prevention of SARS in Beijing [1]. However, it can be seen that Hong Kong and Singapore applied strict school closures; they did have fewer days in peak periods which were around 60 days during the SARS outbreak, and good cured rates as well; all other areas had more days instead in their peak periods, except Taiwan. Taiwan was on the WHO’s list of epidemic areas for the longest time, which was 105 days. Taiwan did not apply school social distancing measures (including closures) and reported the worse cured rate. There is a lack of data to show the impact of school closures in the SARS outbreak, however.
- Canada has the highest HCW infection rate, and the highest mortality rate as well. Toronto was put on the WHO epidemic areas list twice. Some related articles only compare mortality rates between countries and/or regions or mention limitations on access to medical services in Toronto; however, those works have not examined the underlying reasons [2,4,5].

In Table 6, from four features in Section 3, we compare and finalise five areas’ final peak periods, present in Table 7. (The event timeline feature is not counted here since these are related to peak periods, not the entire outbreak being contained as the WHO announced.)

From the discussions above, we believe that case data from Hong Kong and Singapore are the most comprehensive, and come with fewer issues (issues indicate that data do not match from different features). They all used strict social distancing measures, such as school closures, etc., in the SARS outbreak when there was no vaccine (there were no approved antiviral drugs that effectively targeted SARS [49]). Especially in Hong Kong, which was affected by SARS most, the virus outbreak was handled better than other areas; its data and outbreak pattern might be useful for further data analytics in the COVID-19 outbreak in our future work. 

Concerning our hypotheses, we can conclude:

- For H1, features such as peak period and prevention measures are compared in five areas. The peak periods are around 60 days in all countries and regions except Canada, who struggled in May and Jun; they all applied similar prevention measures such as quarantine, frequent hand washing, avoiding crowded places, non-essential activity, closure etc. However, implementation strictness is different, for example, Hong Kong and Singapore closed schools entirely, but Taiwan did not do the same thing at all. Several schools in Toronto with infection cases were closed.
- For H2, facts such as mortality rate, cured rate, outbreak days are compared in five areas. Results show that similar mortality rates occur in most areas except China, with cured rates varying between 70% and 80% and China at 92.2%. Areas with strict isolation measures tend to have higher cured rates, fewer peak periods and fewer days on the WHO’s list of areas with local transmission.
- For H3, authors are all in IT fields, far from the medical expert field, and those graphs do assist us in understanding the SARS outbreak and bringing fresh insights for us. Some interesting facts are discovered; for example, the quarantine’s impacts on cured rate and peak period, the struggling of Taiwan and Canada (which may be caused by misdiagnosis and/or less quarantine etc.), and that data presented conflict in different respects (e.g., case detail analysis in China, due to the data integrity issues). However, we have not conducted a relevant survey to provide data support on it yet.
- For H4, as discussed above, Hong Kong and Singapore could be used as a good reference for SARS lifecycle analysis as they provided complete datasets with less data integrity issues, as well as applied strict measures, and had better outcomes. Yet, at this stage, it is difficult to collect accurate data such as age, gender, household income, population density, ethnicity, commute, etc., back to 2003; hence, human behaviour is not considered in this study. Since Hong Kong has the most cases with more data, we suggest using Hong Kong’s pattern as a reference for future related research.

## 5. Conclusions

Through the experiments, we finalised graphs for visual analysis of the SARS outbreak from five major features. This work is not medical and/or clinical; all outcomes were based entirely on data analysis. Hence, this work is for people who have an interest and addresses final statistical data rather than virology knowledge. We do obtain some insights from the complex raw data via visual analysis, and the visualisation methods could be useful for related research. Since many researchers are interested in COVID-19 studies at this particular period, this work may offer some different views on it. This is also our future work, applying the current research methodology to COVID-19 data analysis, and seeing if we can discover something new in the COVID-19 outbreak from a visual analysis perspective.

## Figures and Tables

**Table 1:** Raw data collected for the SARS outbreak.

Countries and Regions	Case Data Numbers	Events Data Numbers
China	108	12
Hong Kong	117	8
Taiwan	116	3
Singapore	117	8
Canada	117	8
Others	n/a	10

Others in the table indicate the WHO or other areas, for event data only.

**Table 2:** *p*-value of every two areas on to-date mortality rate.

*p*-Value (Alpha = 0.05)						
	Area	China	Hong Kong	Taiwan	Singapore	Canada
Area						
**China**			1.57035 × 10^−16^	5.96062 × 10^−51^	2.22093 × 10^−^^32^	7.25899 × 10^−45^
**Hong Kong**		1.57035 × 10^−16^		**0.460370636**	**0.165184352**	8.21687 × 10^−43^
**Taiwan**		5.96062 × 10^−51^	**0.460370636**		**0.251680885**	2.68275 × 10^−41^
**Singapore**		2.22093 × 10^−32^	**0.165184352**	**0.251680885**		1.60912 × 10^−41^
**Canada**		7.25899 × 10^−45^	8.21687 × 10^−43^	2.68275 × 10^−41^	1.60912 × 10^−41^	

**Table 3:** *p*-value of every two areas on to-date cured rate.

*p*-Value (Alpha = 0.05)						
	Area	China	Hong Kong	Taiwan	Singapore	Canada
Area						
**China**			**0.50222527**	1.69603 × 10^−11^	0.000462712	**0.249488481**
**Hong Kong**		**0.50222527**		1.44916 × 10^−10^	4.65794 × 10^−6^	**0.675979497**
**Taiwan**		1.69603 × 10^−11^	1.44916 × 10^−10^		5.09475 × 10^−27^	1.09219 × 10^−11^
**Singapore**		0.000462712	4.65794 × 10^−6^	5.09475 × 10^−27^		3.64957 × 10^−10^
**Canada**		**0.249488481**	**0.675979497**	1.09219 × 10^−11^	3.64957 × 10^−10^	

**Table 4:** *p*-value of every two areas on the daily changing rate of infected case number.

*p*-Value (Alpha = 0.05)						
	Area	China	Hong Kong	Taiwan	Singapore	Canada
Area						
**China**			**0.292897097**	0.002374384	**0.743646526**	**0.156640279**
**Hong Kong**		**0.292897097**		**0.017908235**	**0.440487983**	**0.509066914**
**Taiwan**		0.002374384	**0.017908235**		0.003852951	**0.135830414**
**Singapore**		**0.743646526**	**0.440487983**	0.003852951		**0.223632039**
**Canada**		**0.156640279**	**0.509066914**	**0.135830414**	**0.223632039**	

**Table 5:** Events during the SARS outbreak. (16 November 2002–05 July 2003).

Date	Event	Weight
16 November 02	China reported the first case	3
10 February 03	China notified the WHO	2
18 February 03	China CDC announced that the pathogen can be identified as chlamydia	1
12 March 03	WHO issued a global alert	4
15 March 03	WHO issued a heightened global health alert	4
22 March 03	Toronto, parts of mainland China, Hong Kong, Taiwan, Singapore, Vietnam added *	4
25 March 03	Singapore started to enforce compulsory quarantine of any infected person	2
27 March 03	Hong Kong and Singapore closed most schools	2
30 March 03	Hong Kong authorities quarantined estate E of the Amoy Gardens housing estate	2
31 March 03	China announced “Atypical pneumonia prevention and treatment technical plan”	3
05 April 03	Singapore announced that school closures would be extended	1
10 April 03	China started daily reports from all provinces on new cases and measures	3
11 April 03	Beijing added*; WHO issued a global health alert	4
16 April 03	WHO named SARS virus	3
20 April 03	SARS was listed as a legal infectious disease in China, the Minister of Health and the Deputy Secretary of the Beijing Municipal Committee were removed from office	2
22 April 03	In Hong Kong, the schools started to reopen in stages	3
24 April 03	In Beijing, elementary and middle schools were suspended for two weeks;Taipei Municipal Hospital Hoping branch was closed.	3
28 April 03	Vietnam removed *	3
07 May 03	China temporarily classified SARS as a Class B infectious disease	1
14 May 03	Toronto removed *	3
22 May 03	In Beijing, high school seniors resumed classes in stages	3
26 May 03	Toronto added *	3
29 May 03	In Beijing, newly diagnosed cases drop to zero for the first time	3
31 May 03	Singapore removed *	3
13 June 03	Parts of mainland China removed *	4
23 June 03	Hong Kong removed *	4
24 June 03	Beijing removed *	3
02 July 03	Toronto removed *	3
05 July 03	SARS outbreak contained (WHO); Taiwan removed *	6

* added means added onto the list of epidemic areas by WHO; removed means removed from the list of epidemic areas by WHO.

**Table 6:** Peak periods and rate stabilising dates in the SARS outbreak.

Countries & Regions	Daily Existing Infected Case Number	To-Date Mortality Rate	To-Date Cured Rate	Daily Changing Rate of Infected Case Number
China	2 April–08 June 68 days	19 May	05 Jun	30 May
Hong Kong	29 March–26 May 59 days	14 May	26 May	16 May
Taiwan	12 May–08 July 58 days	13 May	08 June	03 June
Singapore	23 March–21 May 60 days	12 May	23 May	20 May
Canada	03 March–21 July 91 days	02 May	17 June	11 July

**Table 7:** Statistical analysis of the SARS outbreak.

Countries & Regions	Peak Period	School Closures	Mortality Rate (%)	Cured Rate (%)	Total Infected /Death/Cured	HCW Infected (%)	Days on the List
China	2 April-08 June 68 days	Beijing: 24 April-22 May 28 days	6.6	92.9	5327/348/4951	19	83(most areas) 74(Beijing)
Hong Kong	29 March-26 May 59 days	27 March-22 April 26 days	17	82.5	1755/298/1433	22	93
Taiwan	12 May-08 July 58 days	N/A	10.7	71.4	671/84/507	20	105
Singapore	23 March-21 May 60 days	27 March–09 April 13 days	13.9	86.1	206/32/172	41	70
Canada	03 March-21 July 91 days	N/A (Several schools closed)	17.1	79.7	250/38/194	43	102

Data collected till 11 July; the list is the WHO’s list of areas with local transmission; school closure counts from the first day of closure to the first day of reopening in stages.
